# Writable electrochemical energy source based on graphene oxide

**DOI:** 10.1038/srep15173

**Published:** 2015-10-14

**Authors:** Di Wei

**Affiliations:** 1 Nokia Technologies, Broers Building, 21 JJ Thomson Av., Madingley Road, CB3 0FA, Cambridge, United Kingdom

## Abstract

Graphene oxide (GO) was mainly used as raw material for various types of reduced graphene oxide (rGO) as a cost effective method to make graphene like materials. However, applications of its own unique properties such as extraordinary proton conductivity and super-permeability to water were overlooked. Here GO based battery-like planar energy source was demonstrated on arbitrary insulating substrate (e.g. polymer sheet/paper) by coating PEDOT, GO ink and rGO on Ag charge collectors. Energy from such GO battery depends on its length and one unit cell with length of 0.5 cm can generate energy capacity of 30 Ah/L with voltage up to 0.7 V when room temperature ionic liquid (RTIL) is added. With power density up to 0.4 W/cm^3^ and energy density of 4 Wh/L, GO battery was demonstrated to drive an electrochromic device. This work is the first attempt to generate decent energy using the fast transported water molecules inside GO. It provides very safe energy source that enables new applications otherwise traditional battery technology can not make including building a foldable energy source on paper and platform for futuristic wearable electronics. A disposable energy source made of GO was also written on a plastic glove to demonstrate wearability.

Nowadays the vast majority of graphene based materials are made from GO due to its potential for scalability and dispensability in a wide range of solvents. Humidity sensors have been made from GO^1^,^2^ and an ultrafast response to humidity can be observed^3^. Such ultrafast permeation of water through GO is due to a low-friction flow of a monolayer of water through two-dimensional capillaries formed by closely spaced graphene sheets^4^. It is recently revealed that nano-confined water exists in ‘square ice’ form in graphene nanocapillaries to enable efficient transport of humidity^5^. Although GO is a well-known electronic insulator, it exhibited extraordinary ionic conduction for protons. Numerous reports suggest that proton conduction along nanoscale films is usually higher than in bulk compounds^6^,^7^. This also applies to GO since it has much higher proton conductivity than its bulky graphite oxide. Protons and small cations can propagate through hydrogen-bonding networks along the adsorbed water film^8^. Properties of such high proton conductivity has been applied in fuel cells^9^ and lead acid batteries^10^. Additionally, GO membranes were used in purification of waste water while producing electricity simultaneously^11^.

Another unique property of GO is its generation of protons under humidity^12^, which was also supported by titration experiments of GO with alkaline^13^. Water molecules were reported to generate protons when in contact with GO either through self-dissociation process^14^,^15^ or chemical reactions^12^. The work presented here discussed the feasibility to apply these properties of GO as energy source and write all components (electrodes, electrolyte, charge collectors etc.) on one piece of polymer sheet/paper. Like in the area of organic photovoltaics, by tailoring the work function of the cathode and anode, the energy and open circuit voltage can be optimized. By coating higher work function conductive polymer, poly(3,4-ethylenedioxythiophene): poly(styrene sulfonate) (PEDOT:PSS), GO-Nafion mixture (1:1 volume ratio) and lower work function rGO on insulating substrate, the planar energy source enabled by GO was found to behave like batteries under humidity. It differentiates with all existing battery techniques (lead acid, lithium ion and zinc manganese batteries etc.), where components of electrodes and electrolyte are needed to be encapsulated into a 3D sealed unit.

## Results

### GO battery: structure and operation

Preparation of GO by exfoliation graphite oxide with different oxidant protocols produces complex mixtures of highly oxidized debris that are strongly bound to GO surface^16^. GO in this paper was made from Hummers’ method and purchased from Graphene Square Ltd. After treating with strong base such as KOH or NaOH, a decrease in the quality of oxygen moieties in GO was observed due to the removal of oxidative debris^17^,^18^. Study also shows that the properties of the base-washed GO are independent of the base used and that it contains similar functional groups as those present in the debris but at a lower concentration^19^. Deoxygenation of GO by KOH may be due to the cleaning of oxidative debris from GO and the graphene-like sheets are unaltered by such cleaning process. The rGO in this paper refers to the GO deoxygenated by KOH.

It should be noticed that GO and rGO are intrinsically two different materials. Addition of the alkaline into GO changes its color (from brown to black) and the addition of acid cannot revert the reaction. Fourier transform infrared spectra (FTIR) spectra from supplementary Fig. S1 shows that both aromatic C–H stretching and sp^2^ C = C stretching are enhanced in rGO and it is also reflected in its decrease in resistance (2 M Ohm from original GO coated on the comb-structure silver electrode in contrast to 5 K Ohm from KOH treated rGO at 1 cm distance as shown in Fig. S2).

**Figure 1:**
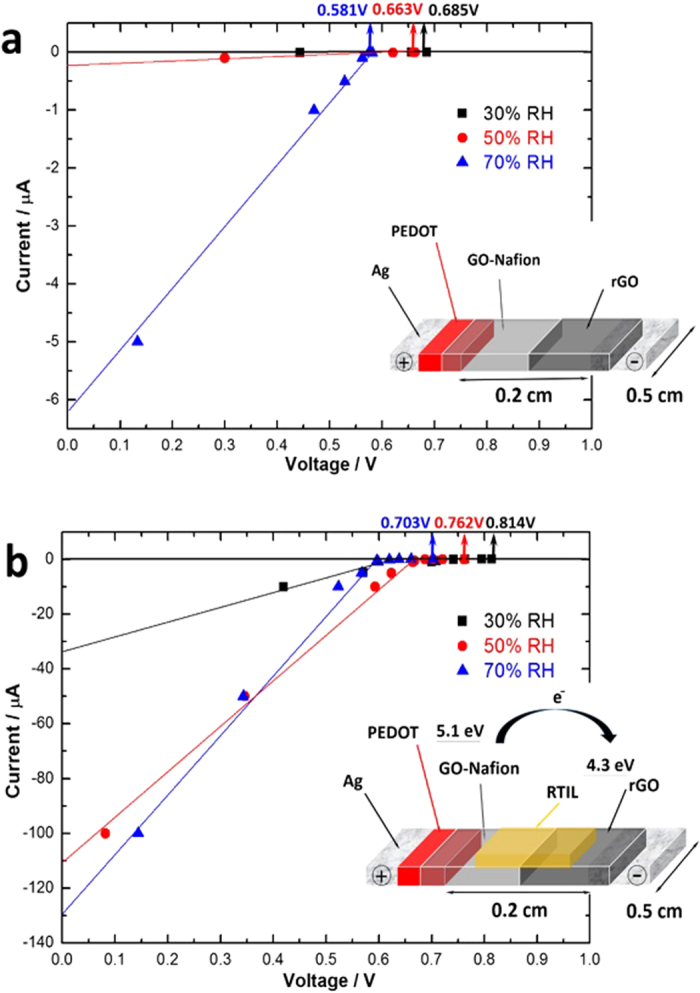
I–V characteristics of PEDOT/GO-Nafion/rGO battery that was coated on polymer substrate of polyethylene naphthalate (PEN) without RTIL (a) and with RTIL (b) under different relative humidity (RH) of 30%, 50% and 70%. Insets: GO battery structure without RTIL (**a**) and with RTIL (**b**).

The planar GO battery is made of PEDOT:PSS, GO-Nafion and rGO as illustrated in Fig. 1. Although battery behaviour was observed using pure GO without Nafion, the hydrophilic nature of GO will easily cause the conductive rGO ink to run into it and short-circuit the charge collectors. As Nafion is a polymer with high proton conductivity and it does not react with GO^20^, it was used to tailor the viscosity and hydrophobicity of GO, allowing creation of sharp junctions using drop-casting or printing techniques (see supplementary Fig. S3 for details). I–V characteristics were studied for GO battery made from pure GO/rGO and GO-Nafion/rGO under different humidity levels (Fig. S4). Addition of Nafion decreases the internal resistance of the GO battery and gives higher capacitive currents at higher humidity levels. The GO batteries presented in the remainder of this paper are made from GO-Nafion 1:1 (volume ratio) solution, which is the optimized ratio to generate largest energy capacity as shown in Fig. S5. Higher ratio of Nafion will reduce the energy capacity of the GO battery. GO has super-permeability to water and this may enable it transport hydrated ions besides protons more efficiently due to the low-friction flow of a monolayer of water through two-dimensional capillaries formed by closely spaced graphene sheets. A room temperature ionic liquid (RTIL) with relatively high conductivity, triethylsulfonium bis(trifluoromethylsulfonyl)imide, was chosen to add on top of both GO-Nafion and rGO coatings. It can be seen that both GO batteries, without RTIL (Fig. 1a) and with RTIL (Fig. 1b) are sensitive to humidity. With increase in relative humidity (RH), the open circuit voltages (as marked in Fig. 1) drops from 0.685 V (RH 30%) to 0.581 V (RH 70%) in Fig. 1a and from 0.814 V (RH 30%) to 0.703 V (RH 70%) in Fig. 1b. Since charge transfer resistance of the GO will decrease with increase in humidity as Fig. S6 shows, decrease in Voc may be due to the increase in conductivity of GO at higher humidity that would cause some short-circuit-like effect as GO is in direct contact with the conductive rGO and PEDOT. However, on the other hand, the short circuit current increases with increase in humidity when more water molecules permeate into the nanopores in the GO battery. Due to the electrically insulating property of GO, the internal resistance of the GO battery is relatively high particularly at low humidity as shown in Fig. 1a. For example, at RH 30% the short circuit current is negligible. It increases to ca. 0.25 μA at RH 50% and jumps quickly to ca. 6.2 μA at RH 70%. It was reported that GO sheet is conductive to electrostatic charge under high humidity (RH >= 50%)^21^. Such charge migration may also contribute to the current in GO battery.

Addition of RTILs significantly boosted the current, power and energy capacity of the GO battery (Fig. 1b). RTILs are molten salts with a melting point close to or below room temperature^22^. Unlike organic solvents, the high boiling point of the RTIL makes the electrolyte never evaporate away during testing. An RTIL with high conductivity, triethylsulfonium bis(trifluoromethylsulfonyl)imide, was chosen to add on top of both GO-Nafion and rGO coatings. Figure 1b shows the short circuit current is increased by hundreds times when the RTIL is added under RH 50%. The open circuit voltage also increases slightly by over 100 mV at all three different RH levels (30%, 50% and 70%). RTIL may have two roles here; firstly it may increase ionic conductivity which contributes the significant improvement in current. Secondly RTIL influences the work function of graphene materials^23^ and the ionic layer may reduce the work function of the rGO^24^, which will increase the open circuit voltage of GO battery.

The higher the humidity level, the more water permeates through the GO flakes. Highest power can be obtained by PEDOT/GO/rGO structure under RH 70% (Fig. 1b). Maximum of 20 μW can be obtained within 0.5 cm * 0.2 cm area (0.1 cm^2^). This translate to power density of about 0.4 W/cm^3^.

### Electrochemical characterization

**Figure 2:**
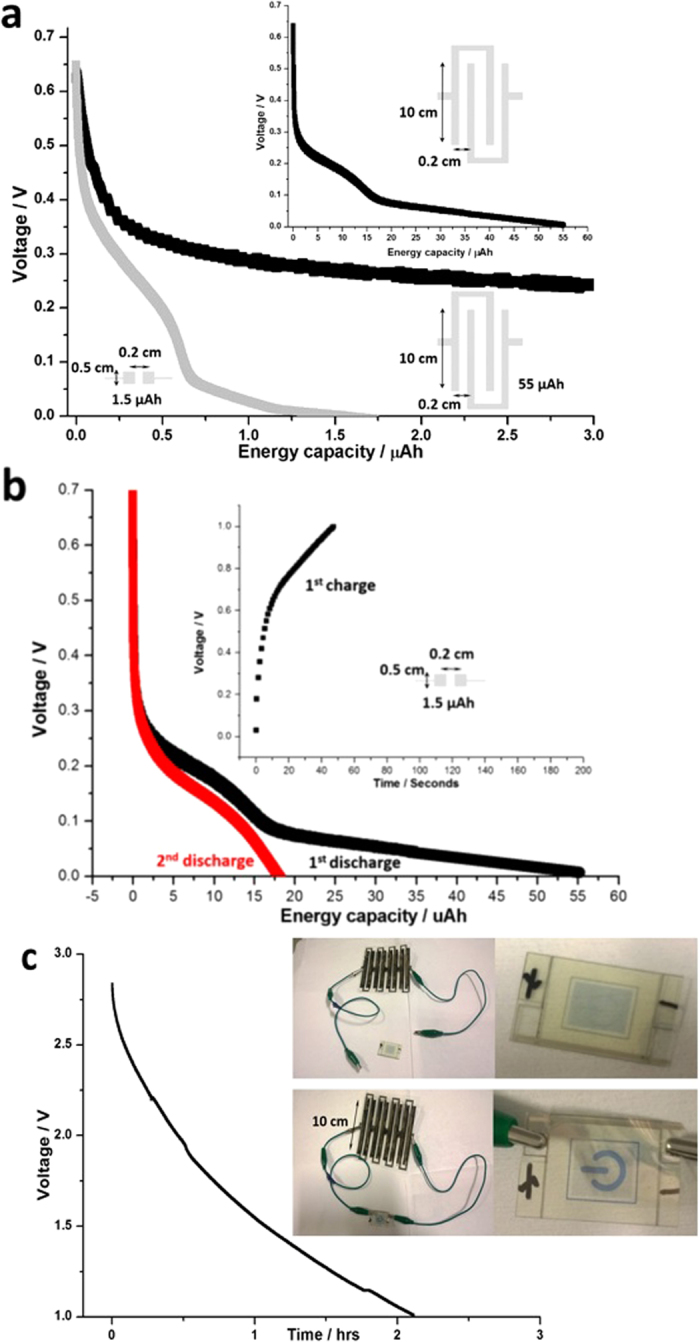
(**a**) Discharge curves for GO battery with different lengths at RH 70%. The discharge current is 1 μA. Inset picture shows the full discharge curve of GO battery with 2 * 10 cm length. (**b**) The first and second discharge at 1 μA and inset picture shows the first charge at 100 μA for GO battery with 2 * 10 cm length at RH 70%. (**c**) Discharge curve of GO batteries printed in series on PEN substrate at ambient environment. The discharge current is 1 μA at ambient environment.

The energy capacity of the GO battery can be scaled up by increasing its length. Figure 2 shows the discharge curves for GO batteries with different lengths.

The energy capacity was improved from 1.5 μAh to 55 μAh, as the length increases from 0.5 cm to 20 cm shown in Fig. 2a. Taking the width of the gap between two Ag electrodes as 0.2 cm, the sheet energy density of the 20 cm-length GO battery is about 0.14 Ah/m^2^, which is similar to energy capacity of the GO battery from 0.2 cm * 0.5 cm area (0.15 Ah/m^2^). Considering the thickness of GO film is about 5 μm, the volumetric energy density of the GO battery is about 0.03 Ah/cm^3^ (30 Ah/L). The volumetric energy density for such GO battery is 4 Wh/L, which is a comparable and even higher than the energy densities from some of the commercial thin film lithium batteries (0.9 to 18 Wh/L)^25^. Surprisingly such GO battery is rechargeable at high rate. After the first discharge of the GO battery at 1 μA (0.67 C rate), it was charged at 100 μA (67 C rate). Within 1 minute, the voltage was saturated at 1 V. Figure 2b shows the second discharge curve at 0.67 C after the 1^st^ charging process (Inset in Fig. 2b) at 67 C rate. Such charge/discharge behaviour may be due to the efficient migration of hydrated cations (e.g. proton and potassium cation from KOH) and the negative charge of GO. To study the rechargeable behaviour, one 0.2 cm * 0.5 cm GO battery was tested under ambient environment by a professional Maccor battery tester. Fig. S7a in supplementary materials shows that GO battery at ambient environment can endure 15 full cycles of discharge and charge at 1 μA between 1 V and 0.01 V. Both energy capacity and the open circuit voltage decreases with increase in discharge cycle numbers. After 15 cycles, the energy capacity of such GO battery drops down to nearly zero. Discharge and charge cycles were repeated 100 times and changes of the energy capacity was shown in supplementary Fig. S7b.

Even taken it as a primary battery, the voltage of the GO battery can be boosted simply by printing several cells in series, allowing it to power electronic devices such as the electrochromic display shown in Fig. 2c. The threshold voltage of the electrochromic device is 1 V and can be powered continuously by 1 μA current. Four series of GO battery units were printed on polyethylene naphthalate (PEN) polymer substrate. It was discharged at 1 μA and the cut-off voltage is 1 V. It can be seen that it will power the electrochromic device for over 2 hours.

### Paper battery and wearable energy source

With expanding interests in wearable electronics, there is keen request to develop reliable wearable energy source to cope with the new form factors. Paper is an inexpensive, lightweight, disposable (environmentally friendly) and foldable substrate and has been demonstrated as promising potential platform to many electronics such as paper diagnostic devices (disposal sensors)^26^, low-cost wireless sensors (RFID tags) and recently even interactive posters made of capacitive sensors embedded in^27^. All previous trials on applying paper in a battery are limited by using paper as matrix to hold electrolyte within the 3D sandwich structure for lithium ion batteries and supercapacitors^28^. The GO battery developed here on the other hand will enable the whole energy source written on one piece of paper directly. Nafion mixed in the GO prevents the conductive PEDOT and rGO inks mixed with the GO coating to enable all components of GO battery to be written on paper. RTILs can be soaked into the whole paper substrate to facilitate efficient ionic conductivity to boost the voltage of GO battery.

**Figure 3:**
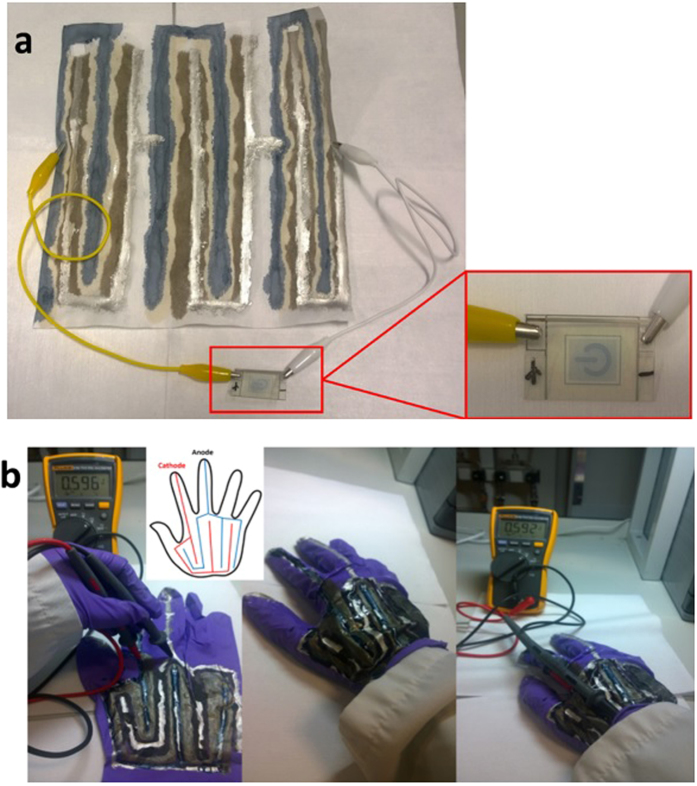
(**a**) Three units of GO batteries written onto a piece of paper to power an electrochromic device. (**b**) GO battery written on glove (Inset picture shows design of electrodes on glove). Both experiments (**a**,**b**) are conducted at ambient environments.

The whole procedure of writing a GO battery on paper is described in Fig. S8 of the supplementary material. Three units of GO batteries written on paper gave a voltage of 1.6 V, enough to turn on the electrochromic device as shown in Fig. 3a. The gap between PEDOT and Ag paint on the paper is over 2 cm. Increase in the gap between the electrodes will increase internal resistance and this may result in the lower open circuit voltage comparing with the ones measured in Fig. 1 at specific 0.2 cm gap between Ag pads printed on PEN substrate. However, the GO battery written on paper follows the basic voltage divider rule. One unit cell of the GO battery written on paper is about 0.5 V. Two units of the GO batteries in series give voltage over 0.9 V as show in supplementary Fig. S9.

The interfacial stress relief capacity of GO and rGO is very strong and this enables it to be coated on elastomeric substrate as well. GO energy source was also coated on the glove to show wearability as shown in Fig. 3b. Inset picture is the design of such unit cell on glove. It can be seen that the voltage of ca. 0.59 V is unchanged even when the glove was worn. Mechanical flexibility is challenge for lithium ion batteries due to the formation of brittle solid electrolyte interface (SEI) layers^29^. The GO energy source does not involve with SEI formation and can be fully flexible. More importantly such safe energy source can be printed for example, by ink-jet printing technology or simply coated onto any insulating substrates.

## Discussion

The exact chemical structure of GO is still a matter of debate: the widely-used *Lerf-Klinowski model*^30^ and the *Szabo-Dekany model*^31^ struggle to explain the high acidity of GO aqueous solutions (pH ~ 3). A new *dynamic structure model* (DSM)^12^ proposes that the acidity of GO originates not from the dissociation of pre-existing acidic groups (their content is very low) but rather by the generation of protons via constant reactions with water. It has also been reported that water molecules generate protons through a self-dissociation process when in contact with GO^14^,^15^. Cations migration such as protons may play an important role in the mechanism of the GO battery.

In the GO battery, the work function of PEDOT (5.1 eV) is higher than rGO (4.3 eV). The difference of work function between cathode and anode will contribute in determination of the Voc value. The current of the GO battery may be determined by the amount of ions/electrons migrated between electrodes during discharge. It should be noticed that pure Nafion can not provide as high energy capacity as GO-Nafion 1:1 (volume ratio). The super-permeability to water of GO may enable it transport hydrated ions besides protons more efficiently due to the low-friction flow of a monolayer of water through two-dimensional capillaries formed by closely spaced graphene sheets. Hydrophobicity of Nafion will also ensure coating fine junctions of the GO battery and realize writting all these battery components on a piece of paper. It is anticipated that protonation happens in GO under humidity or with RTIL coating that provides both improved ionic conductivity and relatively fixed humidity at ambient environment due to absorbed water. Supply of electrons which migrate into the rGO, due to the interface potential gradient caused by the work function difference and a corresponding cations migration such as protons or other hydrated cations may happen inside GO. Theoretical^32^ and experimental^33^ investigations have shown that GO has excellent proton conductivity and negligible electron conductivity, whereas rGO conducts electrons but not protons, enhancing the charge separation and transport.

**Figure 4:**
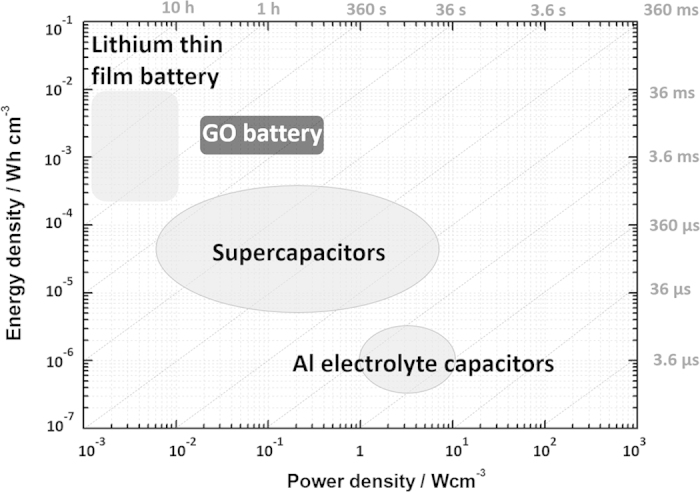
Comparing energy and power density of GO battery in the Ragone plot (Data for lithium thin film battery are collected from ref. 36,37 as 4 V 500 μAh lithium thin film battery and data for supercapacitors and Al electrolyte capacitors are collected from ref. 34,35).

To demonstrate the overall performance of the energy source based on GO, a Ragone plot is shown in Fig. 4. If we compare the energy and power density of GO battery with those reported for supercapacitors^34^,^35^ and lithium thin film batteries^36^,^37^, GO battery has comparable energy density but much higher power density than the reported lithium thin film battery as shown in Fig. 4.

It is known that protons from hydrolysis of GO can move with the assistance of water and contribute to the capacitance in electrochemical energy storage devices made of GO^32^,^38^. According to the ‘Charge Close-Packed model’ (CCP) model^32^, the charge separation is significantly enhanced within the GO film because of the existence of more O and OH species adsorbed on the graphene sheets and the GO layer will attract cations while repel the oxygen with negative charge in the water molecules. These will result in more ordered water molecules confined within nano-gaps of GO film depending on the amount of O and OH species attached to the graphene sheets. The formation of the ordered extraordinary proton conductivity of GO within these nano-gaps doesn’t need a long-range diffusion and may contribute to the extraordinary cation conductivity of GO. More importantly, it has also been demonstrated by Geim *et al.* that smaller ions permeate through the GO membranes at rates thousand times faster than what is expected in simple diffusion and ultrafast molecules (hydrated radii < 4.5 angstrom) sieving through GO membrane is enabled by a network of nanocapillaries that attributed to a capillary-like high pressure acting on the hydrated ions inside^39^. In this model, GO is functioning similar to the biological membranes involving movement of cations (typically K^+^, Na^+^ and proton). This will result in formation of electrochemical gradient due to the movement of cations. Effect of concentration cells and Faradaic reactions involved with migrated cations may contribute to the energy and voltage of the GO battery. With super-permeability to water, proton generation capability and extraordinary proton/hydrated ion conductivity, GO provides a novel energy source as reported in this paper.

In summary, all current battery technology is based on electrodes as storage of ions (e.g. lithium ions in batteries and protons in fuel cells) in a 3D packed cell. In the planar GO battery reported here, the work function difference between PEDOT and rGO may contribute to the open circuit voltage and GO may be the dynamic source to generate protons assisted by the presence of water when in contact with rGO simultaneously functioning as an efficient conductive media for transportation of hydrated cations. Such energy is generated through a lithium-free sustainable battery chemistry process under humidity. Freshly coated GO battery without addition of RTIL needs activation from humidity. In the supplementary material there is a video that shows charging an electrochromic device when water vapour was sprayed on GO batteries. Water evaporation is the largest power source in nature. In fact, simply increasing the humidity from that of a dry, sunny day to a humid, misty one enabled the generation of energy. In this sense, GO energy source can potentially monitor the change in humidity as self-powered sensor as well because higher current will be generated at higher humidity levels. As energy harvester, it generates electricity under humidity with higher energy density than those from thermoelectronics and piezoelectronics. When encapsulated with fixed humidity level, it can be working like an energy storage device and outperforms supercapacitors and some of the thin film lithium ion batteries. As a kind of molten salt, RTIL absorbs water and it can boost the current and power of such GO battery significantly while maintaining fixed amount of humidity on the surface of GO energy source. Paper substrate can absorb the RTIL in an effective way to hold the ionic conductor in the cellulose matrix. The interfacial stress relief capacity of GO and rGO is very strong and it enables GO battery coated on stretchable substrates such as gloves without sacrificing its performance. As GO materials production can be readily scaled up to large quantities, the mass productions of devices can easily be envisaged. The planar battery can be written or printed onto a wide variety of substrates, and manufactured into any shape, which opens the new scenario for the versatile designs of disposable batteries to enable wearable electronics and other complex form-factors.

## Methods

GO solution was bought from Graphene Square Ltd. With concentration of 1 mg/mL. rGO in this paper refers to the GO deoxygenated by KOH. KOH was mixed with GO (originally pH ~ 3) to make a solution with pH 13. Commercially-available 3–4% aqueous dispersion of poly(3,4-ethylenedioxythiophene)-poly(styrenesulfonate) is from Sigma-Aldrich (PEDOT:PSS, Aldrich Prod. No. 655201). The conductivity of the PEDOT film is 150 S/cm with resistance of about 500 Ohm/sq. RTIL, triethylsulfonium bis(trifluoromethylsulfonyl)imide was also bought from Sigma Aldrich and its conductivity is ca. 5.5 mS/cm. Very fine junctions between the coatings of Ag/PEDOT/GO-Nafion/rGO/Ag was observed in the GO battery as shown in Fig. S10. The drop-casting is carried out by a precise syringe (Avanti 1000 μL Syringe) to control the process to ensure same amounts of inks were casted in a given distance.

The humidity was strictly controlled by Voetch environmental chamber. Temperature was fixed at 25 centigrade and humidity (RH) varies from 30% to 70%. Work functions of PEDOT (5.1 eV) and rGO (4.3 eV) were measured by Kelvin Probe (KP Technology Ltd.)

All electrochemical characterization was carried out by Agilent (Keysight) B2902A precision source/measure unit (SMU). Maximum power density of the GO battery can be calculated by the maximum area (I * V) under the slope at 70% RH in Fig. 1b. Maximum of 20 μW can be obtained within 0.5 cm * 0.2 cm GO battery area (0.1 cm^2^). This translates to 0.2 mW/cm^2^ (2 W/m^2^). Considering the thickness of GO film is about 5 μm, which was measured by Asylum AFM under surface profile tapping mode, the power density is about 0.4 W/cm^3^. The energy density was calculated by integration of area of the discharge curves in Fig. 2a. Within the 0.2 cm * 0.5 cm GO battery areas, the stored energy is 0.210 μWh with coating thickness of about 5 μm. This translates to the energy density of about 4 Wh/L.

## Additional Information

**How to cite this article**: Wei, D. Writable electrochemical energy source based on graphene oxide. *Sci. Rep.* **5**, 15173; doi: 10.1038/srep15173 (2015).

## Supplementary Material

Supplementary Information

Supplementary video
