# Supplementary material for: Writable electrochemical energy source based on graphene oxide
Source: Sci Rep. 2015 Oct 14;5:15173. doi: 10.1038/srep15173 (PMC4604454; doi:10.1038/srep15173)
Supplement: Supplementary Information [file srep15173-s1.docx]

*Writable electrochemical energy source based on graphene oxide*

**Di Wei ^*^**

Nokia R&D UK, Broers Building, 21 JJ Thomson Av., Madingley Road, CB3 0FA, Cambridge, United Kingdom

*Email: [di.wei@nokia.com](mailto:di.wei@nokia.com)

Fourier transform infrared spectra (FTIR) spectra of GO, rGO and GO-Nafion.


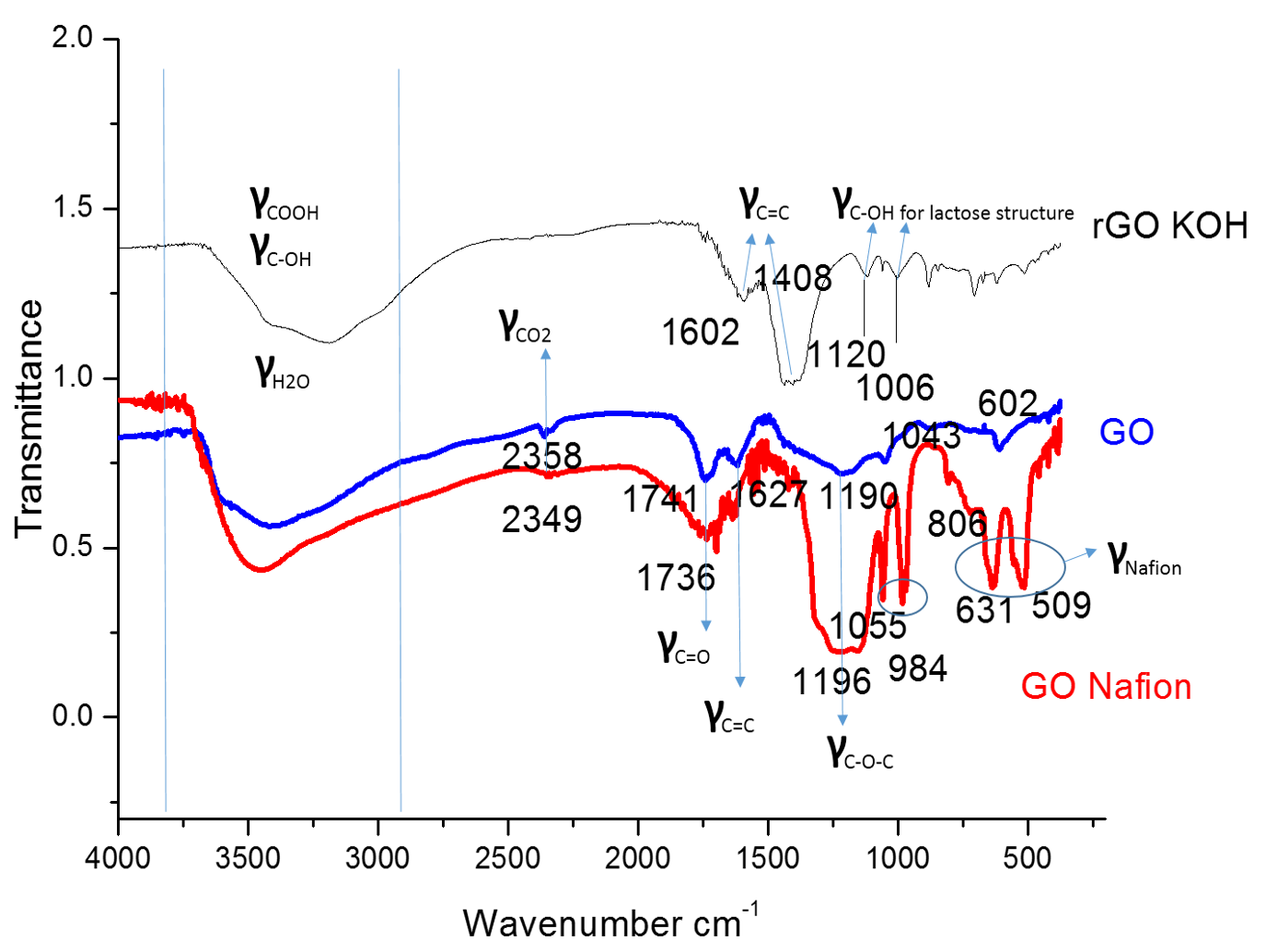


**Figure S1** FTIR spectra for GO, Nafion-GO and rGO.

It shows a very broad feature between ~2500 and 3750 cm^-1^ corresponding to C-OH (C-OH for phenols and lactols at ~3000-3600 and ~1050-1150 cm^-1^), COOH (~1650-1750, ~3000-3600 and ~1050-1200 cm^-1^) and H_2_O stretches ^1^. The band at 3085 cm^-1^ is assigned to aromatic C–H stretching vibration. The stretching mode of the O–H band appears at 3382 cm^-1^. The spectrum also shows several absorption bands in the region between 2000 and 800 cm^-1^, which have been previously assigned to epoxides, alcohols, carbonyls, ketones and sp^2^ carbon ^2^.

IR spectra of GO has intense bands at ca. 1740 cm^-1^ that is assigned to the carbonyl (C=O) stretching mode and the band at 1190 cm^-1^ is due to the C-O-C stretch of the epoxide groups ^3,4^. The phenolic groups ionize to give the phenolate anion (C-O-) that subsequently transforms to the ketone with a characteristic symmetric stretching mode at 1627 cm^-1^ ^5^. In addition to the GO peaks, GO Nafion film also has finger-prints from Nafion polymer and band at 2350 cm^-1^ is from CO_2_.

With further increase in pH, the band at 1740 cm^-1^ decreases in intensity and is absent at pH 13 which was also observed in reference ^3^. The carbonyl (C=O) stretching mode of the undissociated carboxylic (COO) group at 1740 cm^-1^ in rGO disappears. The IR spectra of rGO are dominated by the C=C stretching modes at 1602 and 1408 cm^-1^ of the aromatic segments of the sp^2^ network ^3^. In contrast to GO, the intensities of the oxygen functionalities are significantly weaker, or absent in rGO. It is in accordance with reports on alkaline treatment of GO removing a majority of the oxidized groups ^6-8^. It indicates that epoxy groups have been washed away by alkaline (e.g. KOH) and high content of carboxyls is also not supported by spectroscopy data such as NMR and XPS etc ^9-13^. The carboxyl band at ~1740 cm^-1^ is considerably weaker, whereas the sp^2^-C mode at ~1600 cm^-1^ is stronger. This spectra information suggests an increase of sp^2^ conjugation domain density (sp^2^-hybridized C=C in plane vibration at ~1600 cm^-1^) in the carbon backbone following base treatment ^14^. Both aromatic C-H stretching and sp^2^ C=C stretching are enhanced in rGO.


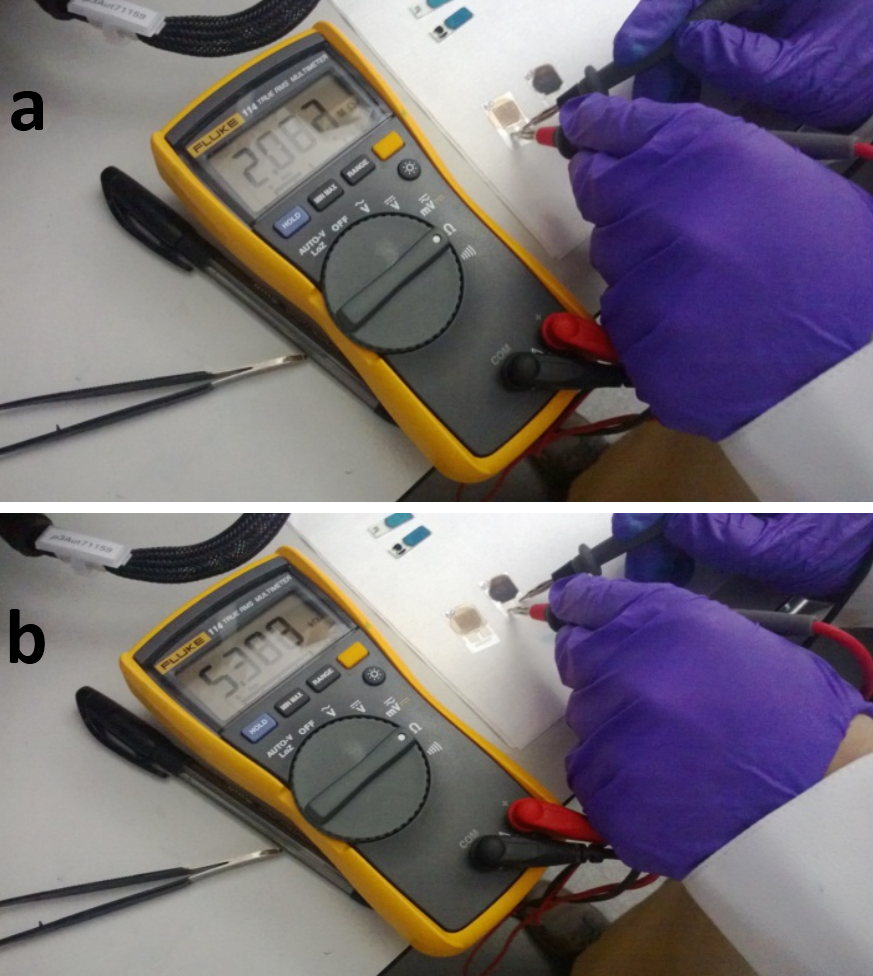


**Figure S2** Conductivity of the original GO coated on the comb-structure silver electrode is 2.062 MOhm (a) in contrast to that from KOH treated GO (b), which is 5.388 KOhm.

Different ratios of GO aqueous solution mixed with Nafion can be made to suit different inks with enhancing hydrophobicity by increasing the ratio of Nafion.

Comparison of coated junction with mixed Nafion and pure GO was as following picture in Figure S3:


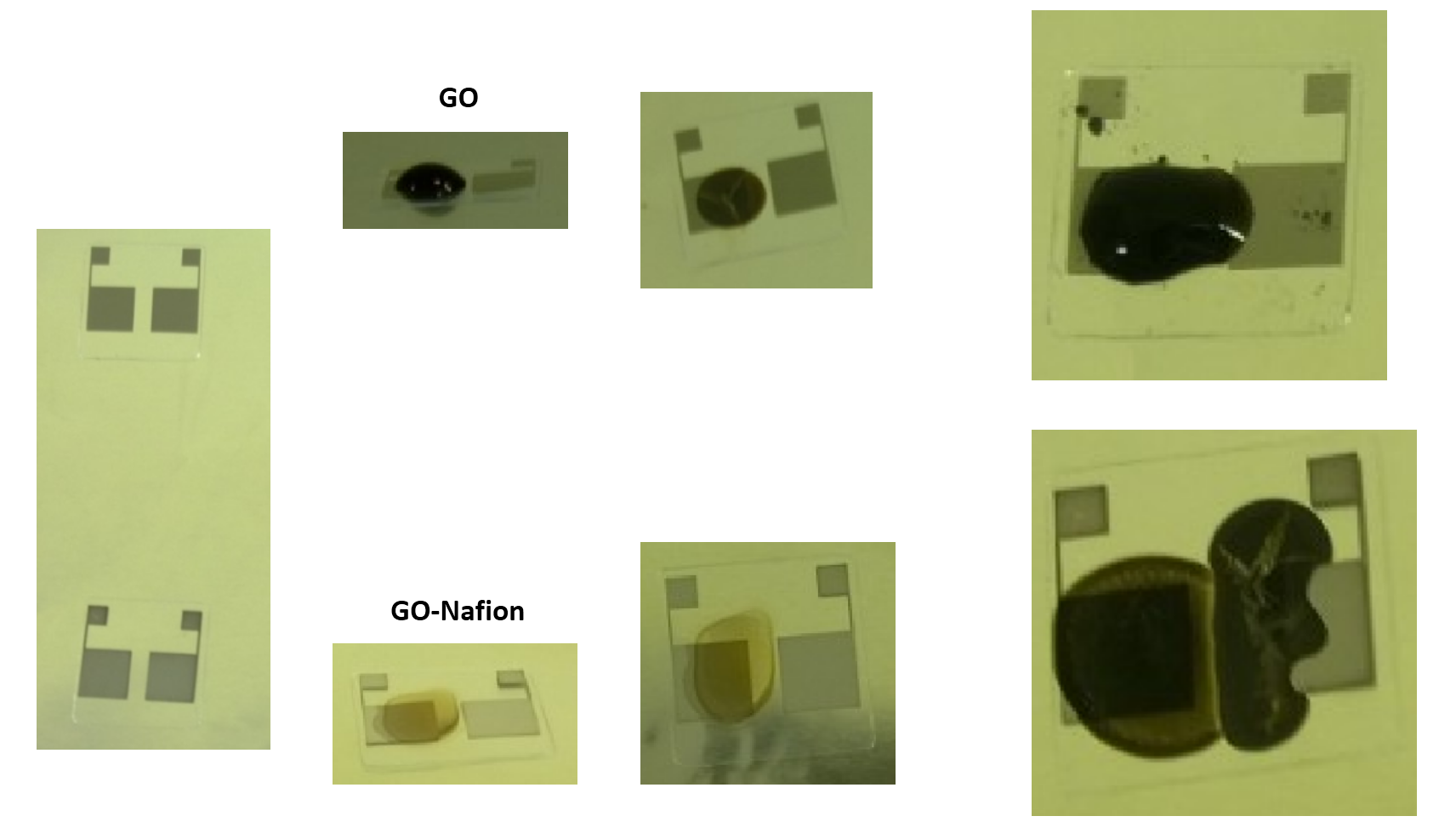


**Figure** **S3** the Nafion modified GO can coat a finer junction.

The hydrophilic GO aqueous solution has strong surface tension on the Ag pad electrode and it is easily overlapping with the rGO ink when coated in adjacent each other within the 2 mm gap of Ag pads. However, coating of GO-Nafion solution and rGO ink can make very fine junction within the 2 mm gap between Ag electrodes.

More effectively, if we printed the pure GO and rGO ink on paper substrate, they will easily immerged into the paper and quickly short-circuit the GO battery. GO-Nafion ink has hydrophobic property and will prevent such short-circuitry to enable a GO based energy source on a piece of paper.


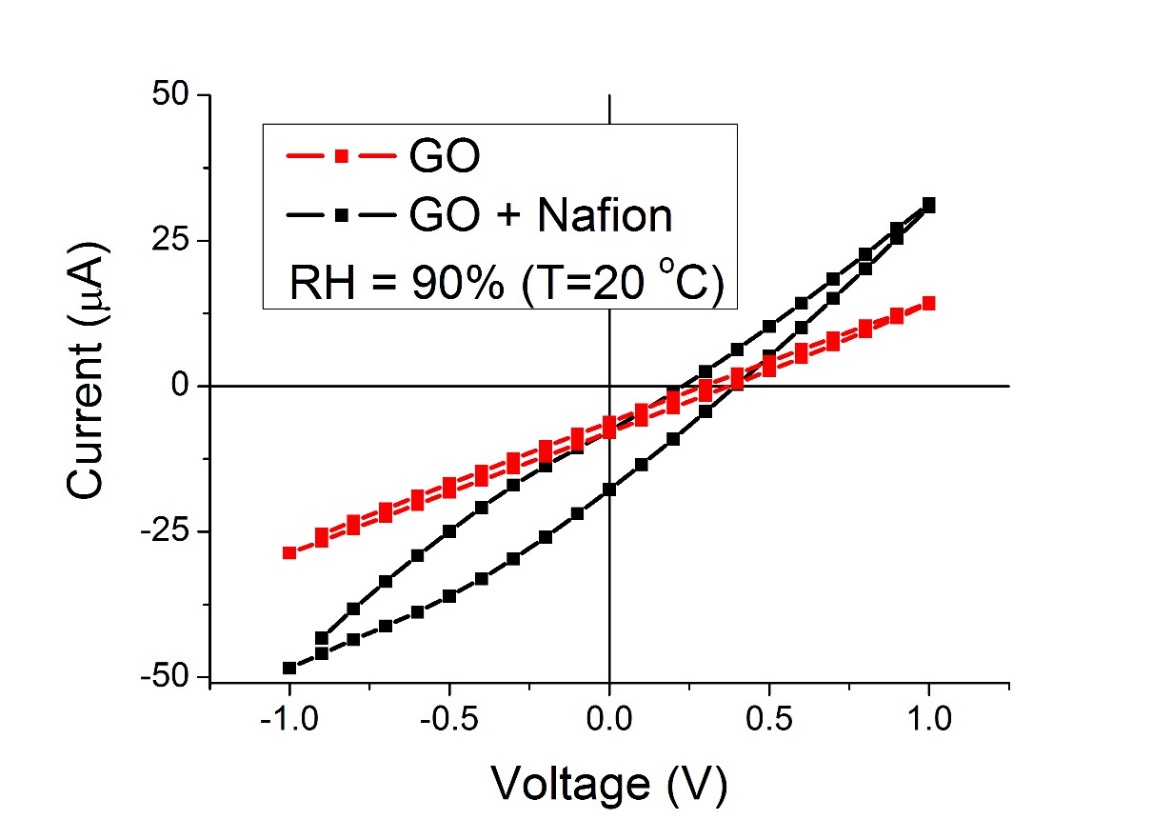

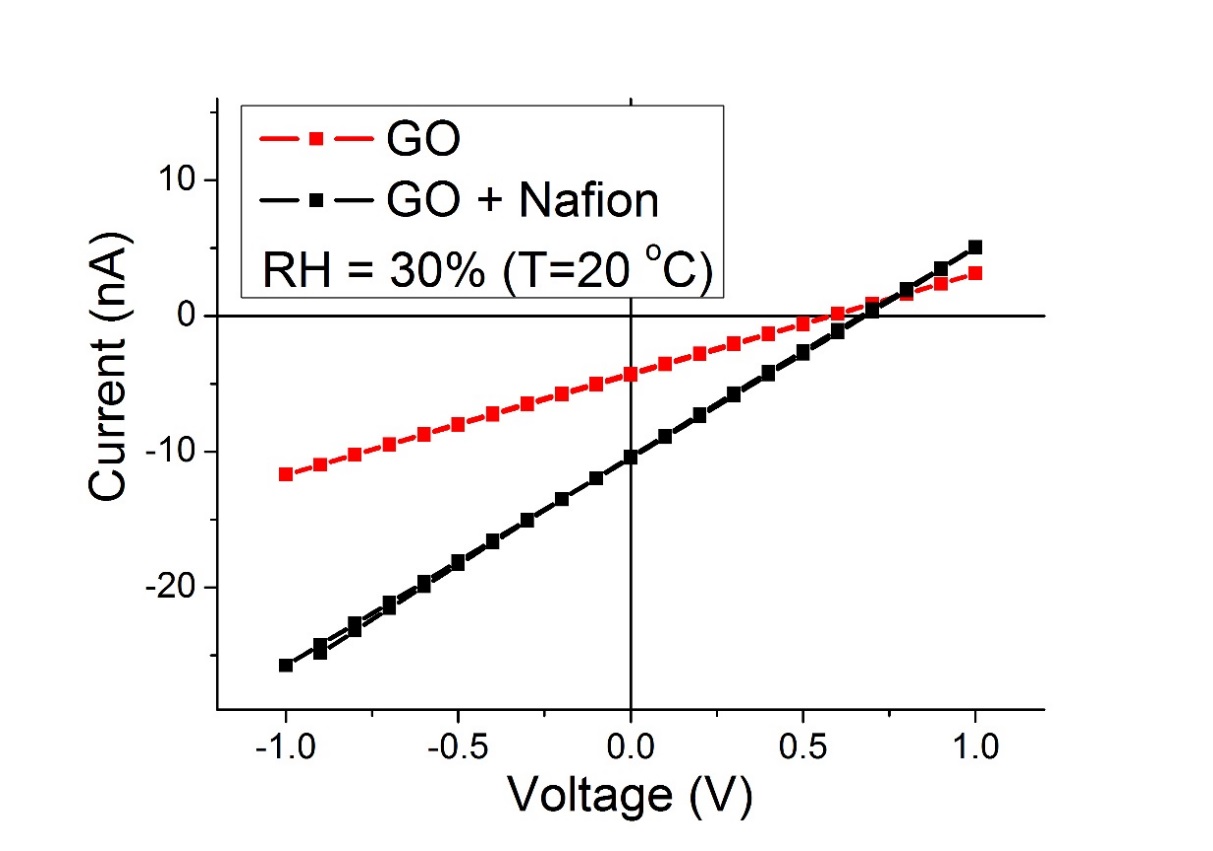


**Figure** **S4** Humidity influence of GO battery made from GO:rGO and GO-Nafion:rGO.

I-V characteristics were studied on pure GO/rGO and GO-Nafion/rGO under different humidity influence (Relatively Humidity 30% and 90%). It shows that addition of Nafion will decrease the internal resistance of the GO battery indicated by the slope in Figure S4. It also gives higher capacitive currents at higher humidity level. However, the open circuit voltage (Voc) is unchanged. It should be noticed that the Voc drops at RH 90%. The decrease in resistance in GO at higher humidity level may partially short-circuit the GO batteries and cause the drop in voltage as shown in Figure S4.


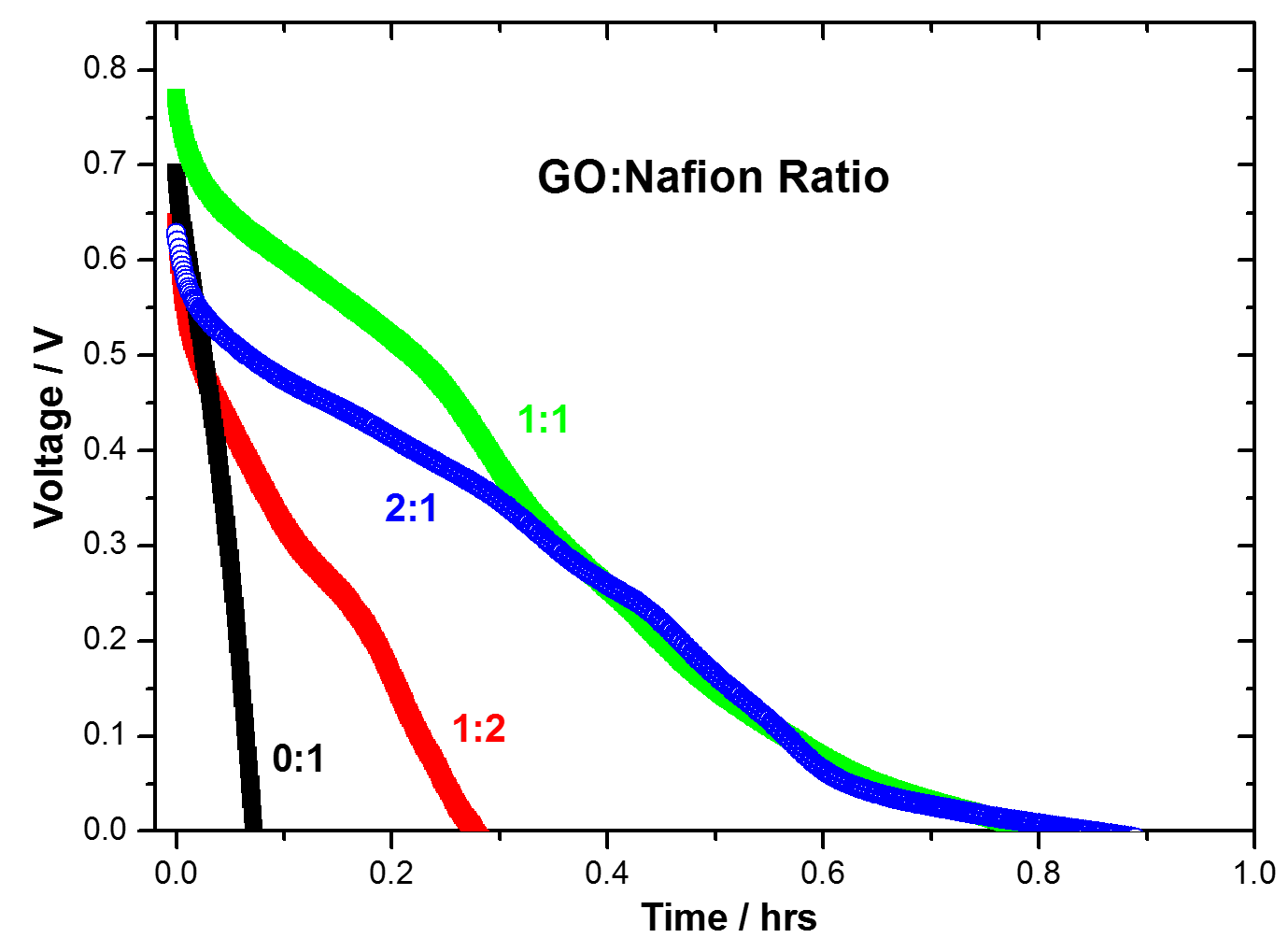


**Figure S5** Discharge curves of GO batterywith different volume ratio of GO:Nafion at 1 uA.

Higher ratio of Nafion will reduce the energy capacity of the GO battery. GO is super-permeability to water and this may enable it transport hydrated ions besides protons more efficiently due to the low-friction flow of a monolayer of water through two-dimensional capillaries formed by closely spaced graphene sheets. However, increase the GO:Nafion ratio from 1:1 to 2:1 does not improve the energy capacity futher. Thus the volume ratio for GO:Nafion is kept at 1:1 in this paper.


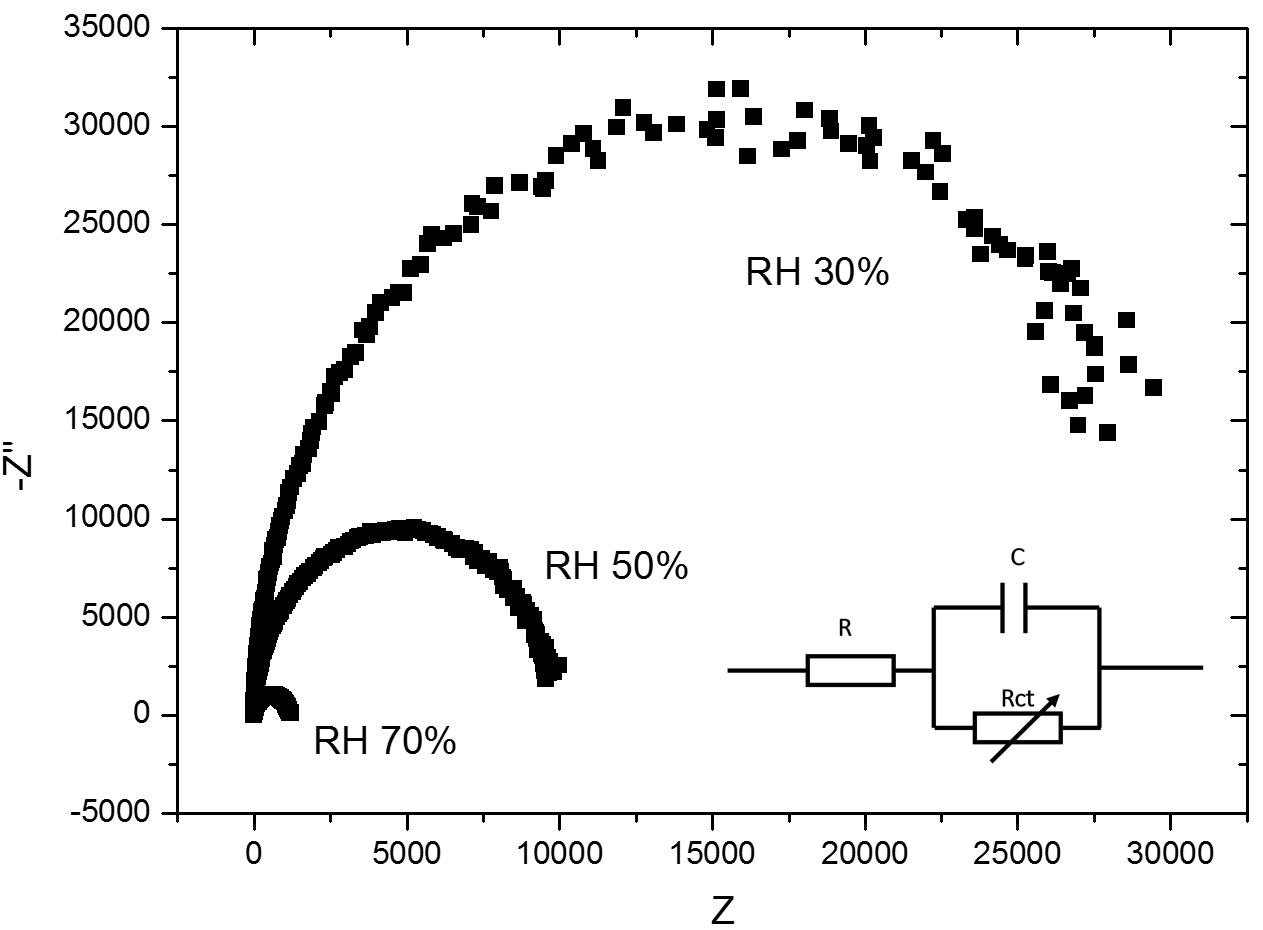


**Figure S6** Nyquist plots of electrochemical impedance spectroscopy (EIS) for the GO battery (Ag/GO/rGO/Ag) under various relative humidity (RH) conditions 30%, 50% and 70% with frequency range of 1 KHz to 1mHz and amplitude of 10 mV.

EIS was performed using an Autolab as shown in Fig. S5. The diameter of the semi-circle in the spectroscopy indicates the charge transifer resistance as Rct represents in the equivalent circuit in the inset of Fig. S5. The charge transifer resistance decreases from about 30 KOhm (RH 30% ), to 10 KOhm (RH 50% ) and to 2 KOhm (RH 70% ). Thus charge transfer resistance of the GO will decrease with increase in humidity.


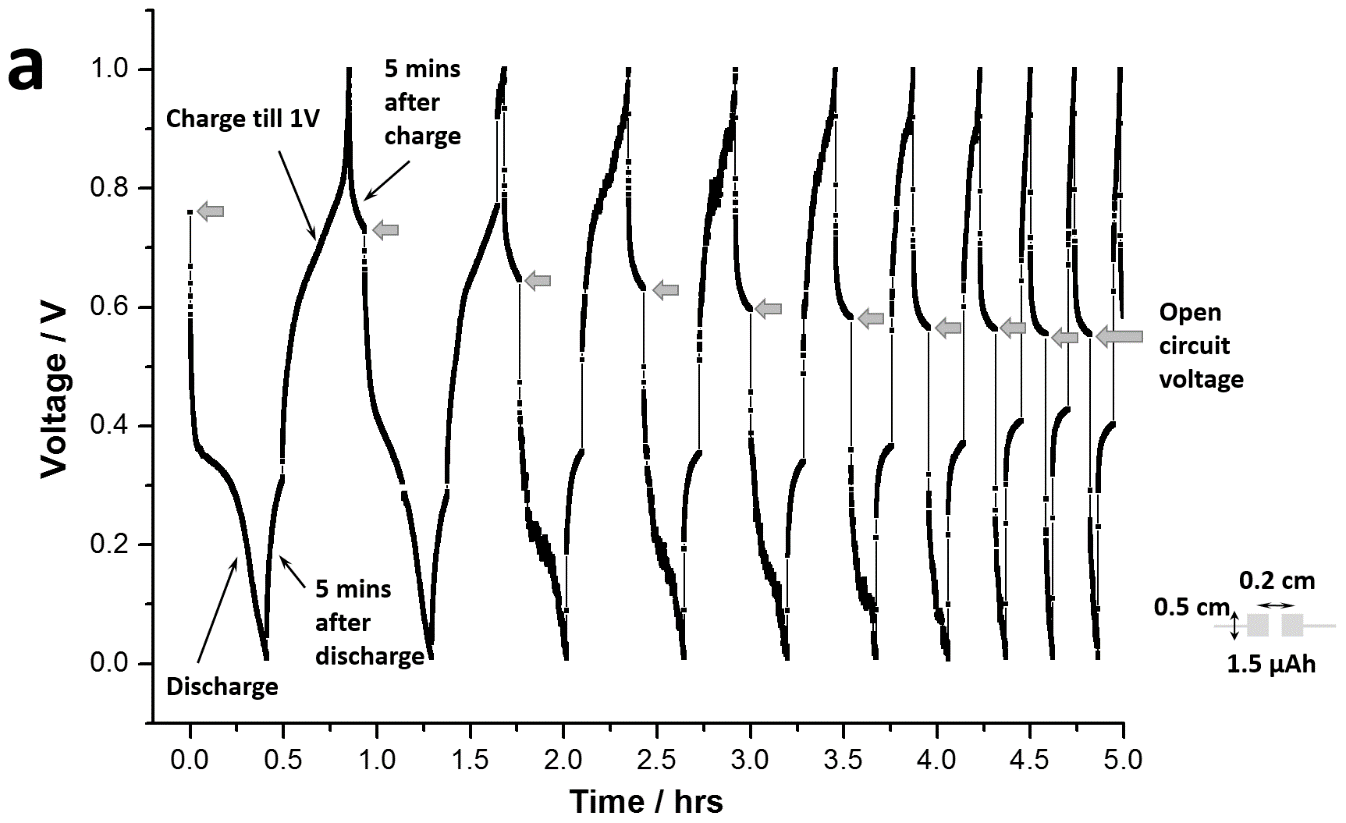


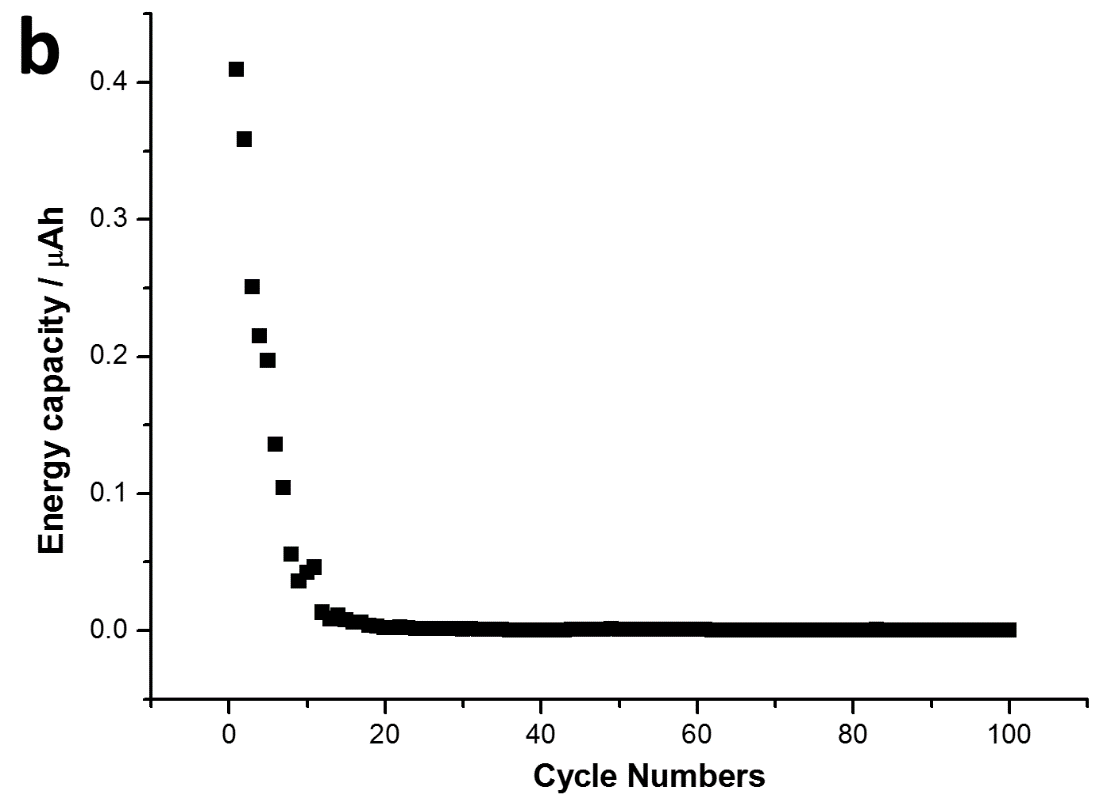


**Figure S7** a) Discharge and charge curves for GO battery (the first 10 cycles) at 1 μA. It was first discharged and then waited for 5 minutes, then charged to 1V, waited for another 5 minutes before the next discharge. b) Change of energy capacity for the first 100 cycles under discharge/charge current at 1 μA. Results are based on one 0.2 cm*0.5 cm (0.1 cm^2^)-area GO battery under ambient environment by a professional Maccor battery tester.


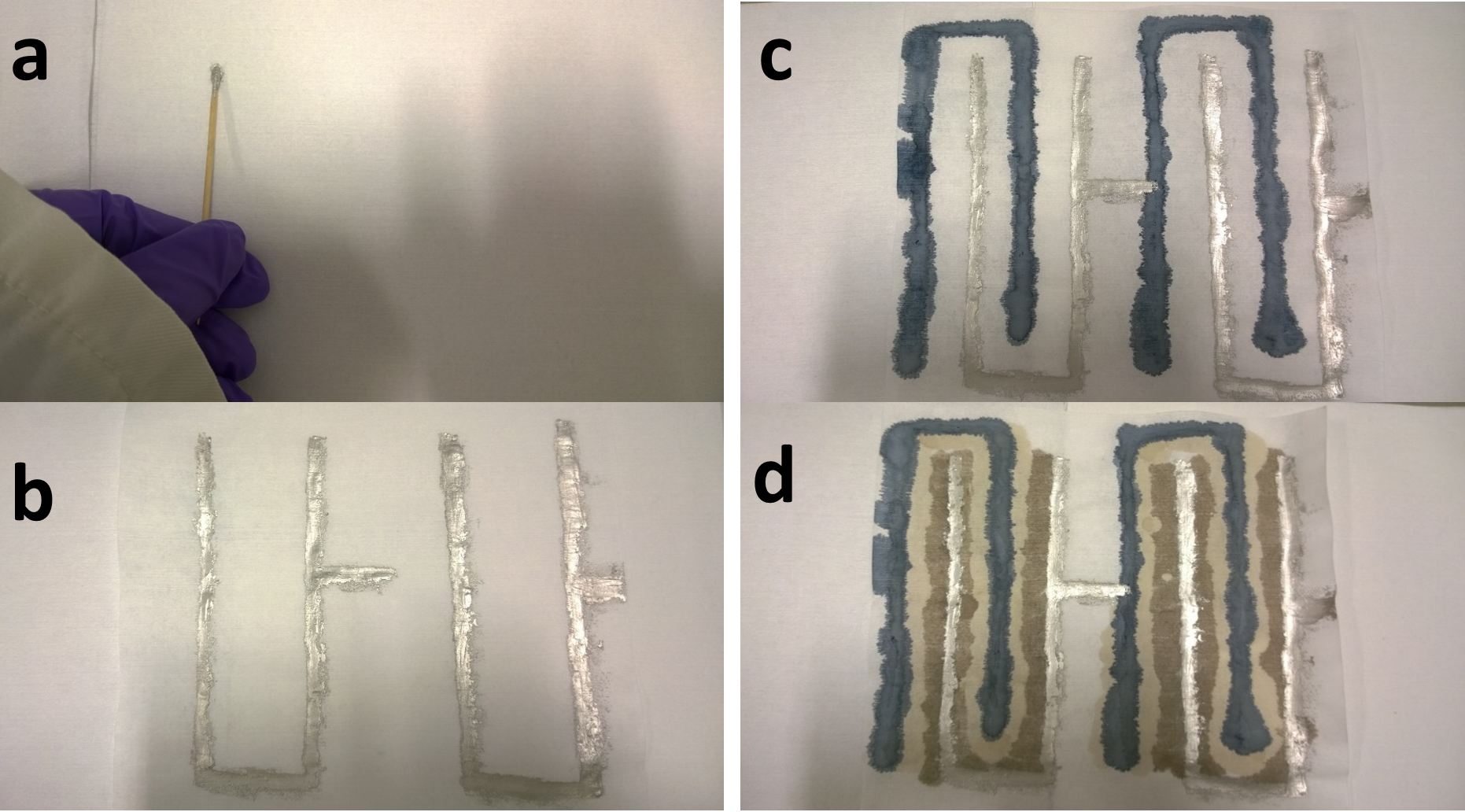


**Figure S8** Write the GO battery on a piece of paper. GO-Nafion can enable clean junction formation on paper substrate and make the printed 2D paper battery feasible.


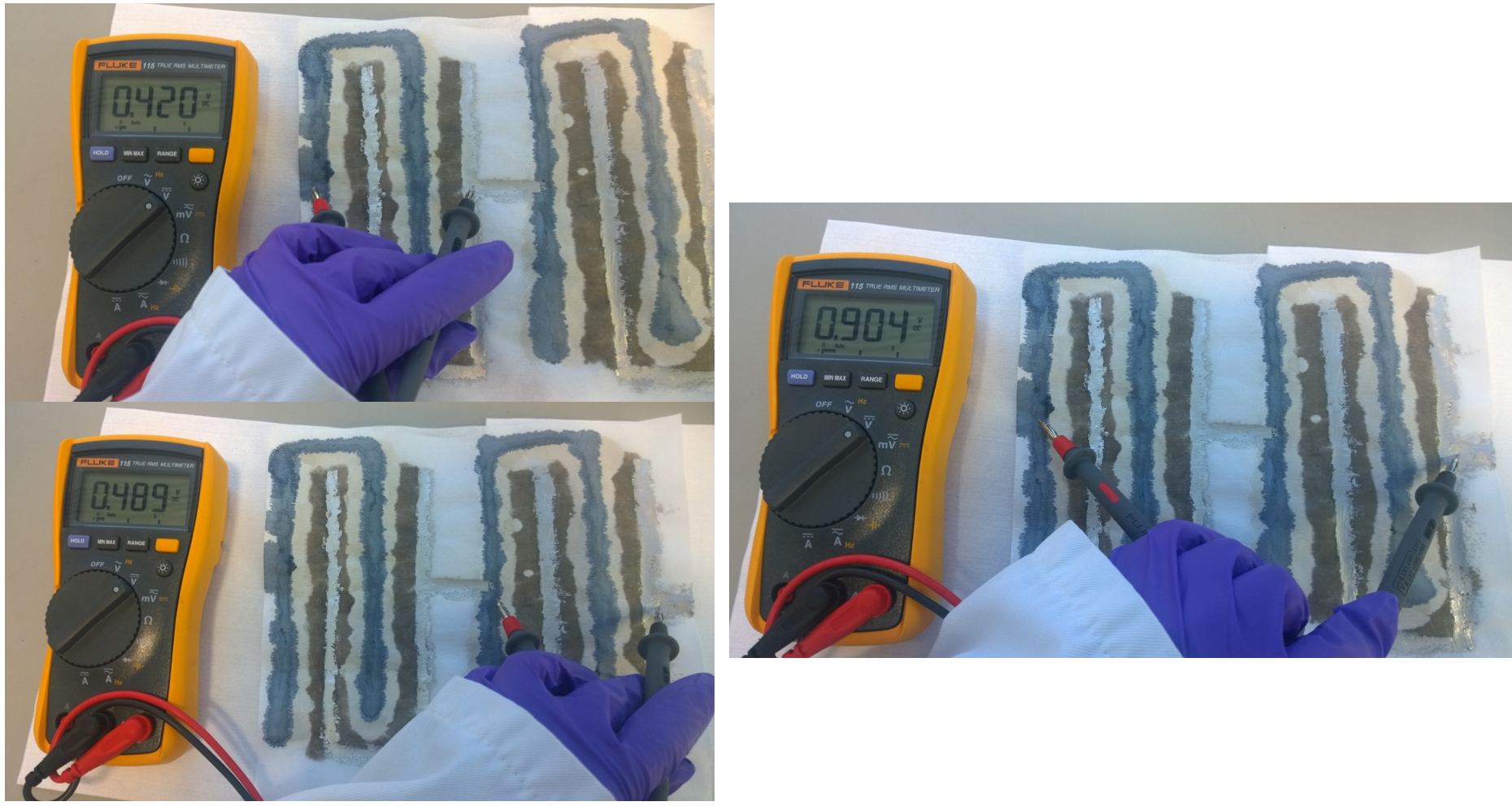


**Figure S9** Two GO paper battery units connected in series. It follows the voltage divider rule.

The gap between PEDOT and Ag paint on the paper is over 2 cm. Increase in the gap between the electrodes will increase internal resistance and this may result in the lower open circuit voltage comparing with the ones measured in Fig 1 at specific 2 mm gap on PEN substrate.


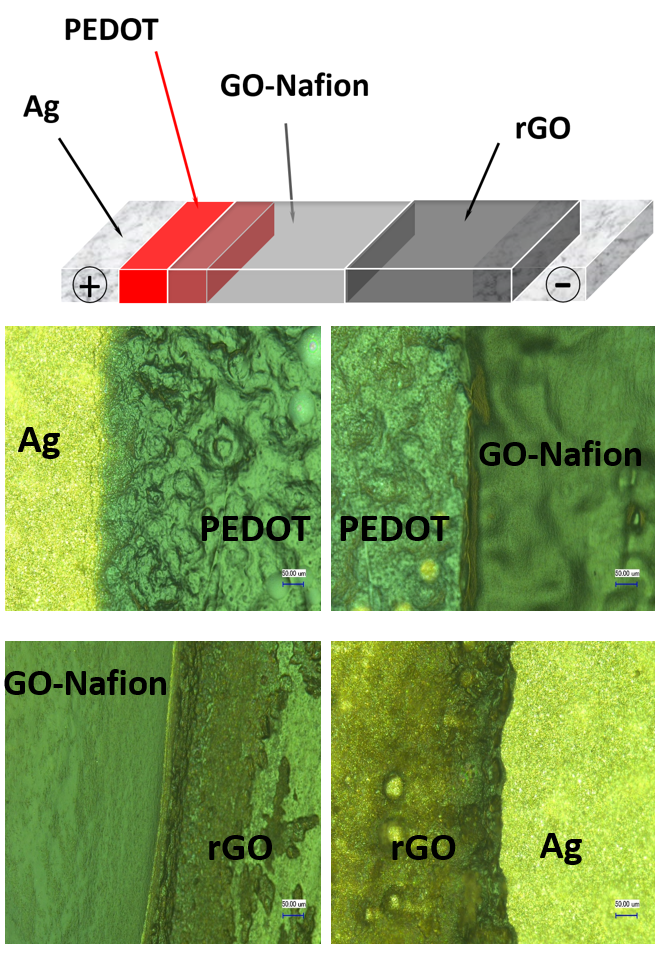


**Figure S10** Digital microscopic picture shows the fine junctions between the coatings of materials in the GO battery.

**The GO battery without RTIL can also be initiated by humidity vapour. A video of charging an electrochromic device when water vapour was sprayed was also supplied.**

REFERENCES

1. O'Neil, G. D., A. W. Weber, R. Buiculescu, N. A. Chaniotakis, and S. Kounaves (2014) *Langmuir* **30,** 9599-9606.

2. Acik, M., G. Lee, C. Mattevi, M. Chhowalla, K. Cho, and Y. J. Chabal (2010) *9* 840-845.

3. Konkena, B. and S. Vasudevan (2012) *J. Phys. Chem. Lett.* **3,** 867-872.

4. Si, Y. and E. T. Samulski (2008) *Nano Lett.* **8,** 1679-1682.

5. Brodskii, A. I., L. A. Kotorlenko, S. A. Samoilenko, and V. D. Pokhodenko (1971) *J. Appl. Spec.* **14,** 633-638.

6. Coluci, V. R., D. S. T. Martinez, J. G. Honorio, A. F. de Faria, D. A. Morales, M. S. Skaf, O. L. Alves, and G. A. Umbuzeiro (2014) *J. Phys. Chem. C* **118,** 2187-2193.

7. Fan, X., W. Peng, Y. Li, X. Li, S. Wang, G. Zhang, and F. Zhang (2008) *Adv. Mater.* **20,** 4490-4493.

8. Pei, S. and H. Cheng (2012) *Carbon* **50,** 3210-3228.

9. Gao, W., L. B. Alemany, L. Ci, and P. M. Ajayan (2009) *Nat. Chem.* **1,** 403-408.

10. Cai, W., R. D. Piner, F. J. Stadermann, S. Park, M. A. Shaibat, Y. Ishii, D. Yang, A. Velamakanni, S. J. An, M. Stoller, J. An, D. Chen, and R. S. Ruoff (2008) *Science* **321,** 1817.

11. Dimiev, A. M., D. V. Kosynkin, L. B. Alemany, P. Chaguine, and J. M. Tour (2012) *J. Am. Chem. Soc.* **134,** 2815-2822.

12. Tang, V., M. Allen, Y. Yang, and R. Kaner (2009) *Nat. Nanotech.* **4,** 25-29.

13. Li, D., M. B. Muller, S. Gilje, R. B. Kaner, and G. G. Wallace (2008) *Nat. Nanotech.* **3,** 101-105.

14. Su, C., M. Acik, K. Takai, J. Lu, S. Hao, Y. Zheng, P. Wu, Q. Bao, T. Enoki, Y. J. Chabal, and K. P. Loh (2012) *Nat. Commun.* **3,** 1298.

15. Ramesha, G. K. and S. Sampath (2009) *J. Phys. Chem. C* **113,** 7985-7989.
